# Clival chordoma with an atypical presentation: a case report

**DOI:** 10.1186/1752-1947-6-410

**Published:** 2012-11-29

**Authors:** Jaber Alshammari, Philippe Monnier, Roy T Daniel, Kishore Sandu

**Affiliations:** 1 Department of Otorhinolaryngology, University Hospital - CHUV, Lausanne, Switzerland; 2 Department of Neurosurgery, University Hospital - CHUV, Lausanne, Switzerland; 3 Department of Otorhinolaryngology, Valais State Hospital - CHCV, Sion, Switzerland

## Abstract

**Introduction:**

Clival chordomas present with headache, commonly VI cranial nerve palsy or sometimes with lower cranial nerve involvement. Very rarely, they present with cerebrospinal fluid rhinorrhoea due to an underlying chordoma-induced skull base erosion.

**Case presentation:**

A 60-year old Caucasian woman presented with meningitis secondary to cerebrospinal fluid rhinorrhoea. At first, radiological imaging did not reveal a tumoral condition, though intraoperative exploration and tissue histology revealed a chordoma which eroded her clivus and had a transdural extension.

**Conclusion:**

Patients who present with meningitis and cerebrospinal fluid rhinorrhoea could have an underlying erosive lesion which can sometimes be missed on initial radiological examination. Surgical exploration allows collecting suspicious tissue for histological diagnosis which is important for the actual treatment. A revision endoscopic excision of a clival chordoma is challenging and has been highlighted in this report.

## Introduction

Chordomas are rare, slow-growing malignant bony tumors that arise from remnants of the notocord and account for approximately 1% of intracranial tumors [1]. Approximately 35% to 40% of these tumors occur in the skull base, where they typically involve the clivus [1-3]. While these tumors rarely metastasize to distant sites, they are locally aggressive and tend to recur after surgical resection, with a life expectancy of less than 10 years after diagnosis [4]. Most commonly, the patient presents with headache, diplopia secondary to VI cranial nerve paresis and visual changes including blurring or sometimes loss of vision [5]. The patient may present with multiple lower cranial nerve palsy symptoms such as facial numbness and asymmetry, dysphagia, hoarseness and speech problems. Finally, large tumors may cause brainstem compression and patients may present with long tract signs and ataxia [6,7]. Epistaxis as a rare presentation has also been reported [8].

Cerebrospinal fluid (CSF) rhinorrhea has been very rarely described as a presenting symptom [9,10]. We present a case report of a patient who presented initially with meningitis and later was diagnosed to have a clival chordoma. We discuss the treatment modalities and present a systematic review of the literature.

## Case presentation

**Figure 1:**
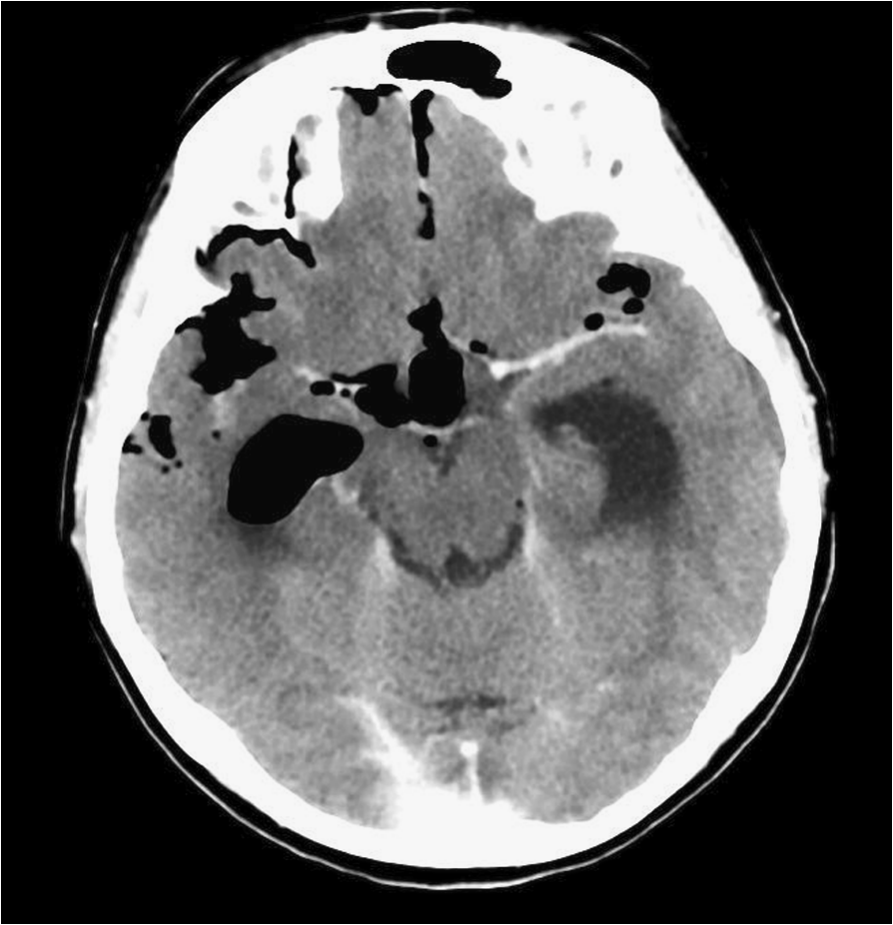
Computed tomography scan showing presence of air in the subarachnoid spaces and ventricles.

**Figure 2:**
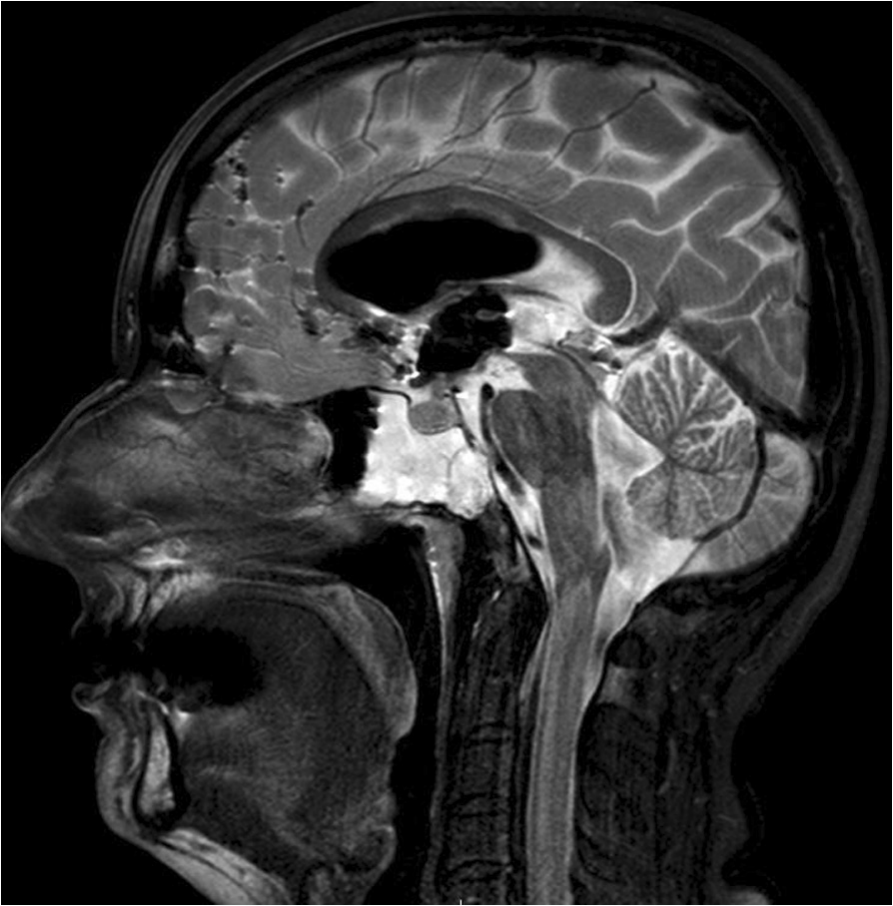
**T2-weighted sagittal images showing erosion of the posterior wall of the clivus and hyperintensity suggestive of cerebral spinal fluid, with a fluid level in the sphenoid sinus.** Note also, the presence of pneumocephalus with air in the subarachnoid spaces and the ventricle.

**Figure 3:**
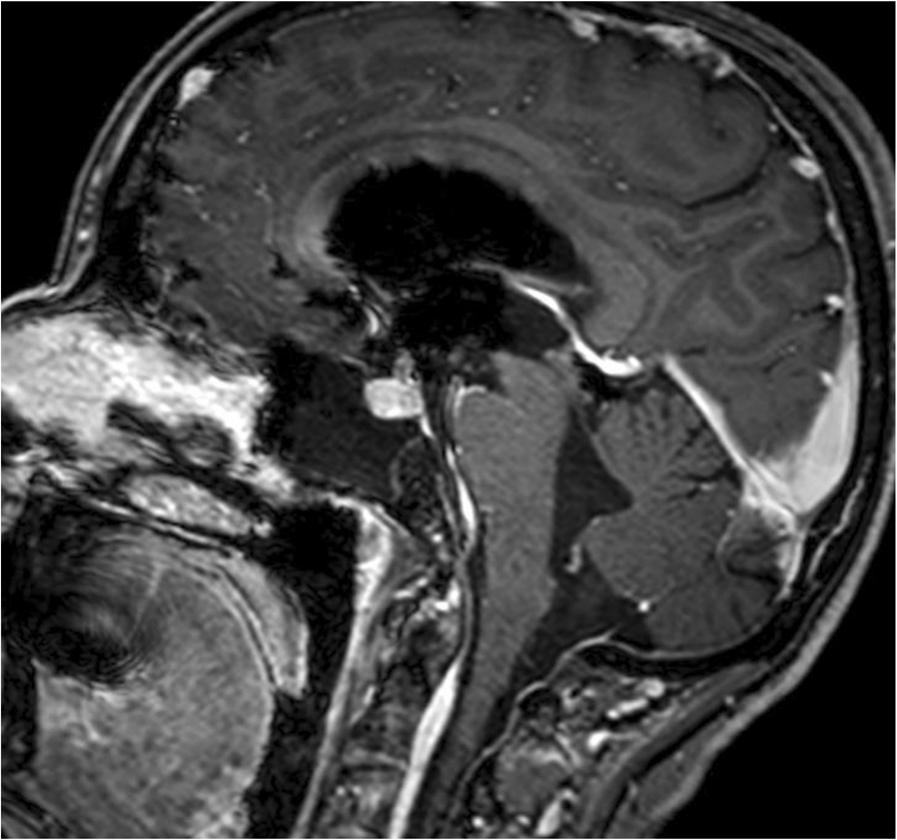
T1-weighted sagittal images showing an osteolytic lesion of the clivus with no contrast enhancement.

A 60-year-old Caucasian woman, living alone and previously in good health, was found unconscious at home and fortunately helped by her friends and the emergency medical team. One week before this incident, she had infrequent episodes of watery nasal discharge that increased with forced sneezing and cough. On her arrival to our clinic, she was confused and febrile. A cranial nerve examination was normal. A computed tomography (CT) scan (Figure 1) of her brain and paranasal sinuses showed pneumocephalus in the ventricles, hydrocephalus and an air-fluid level in the right sphenoid sinus indicating a CSF leak. There was a bony defect of the clivus with sclerotic edges. A lumbar puncture and analysis of her CSF confirmed bacterial meningitis and our patient was immediately started on ceftazidime 2g three times a day. Magnetic resonance imaging (MRI) (Figures 2 and 3) showed an osteolytic, irregular lesion occupying the upper clivus and measuring 13×12×16mm. It was hyperintense on T2-weighted images and hypointense on T1-weighted images. The lesion showed no enhancement with administration of gadolinium. An endonasal transsphenoidal endoscopic exploration of the skull base defect and closure of the CSF fistula was planned. A bony defect and CSF leak was visualized on the postero-inferior wall of her right sphenoid sinus at the posterior cortical margin of her clivus. Further exploration revealed suspicious tissue adjacent to the dural defect that was just anterior to her basilar artery. This tissue was carefully dissected and sent for histopathological examination. The skull base defect was repaired using fascia lata and the sphenoid sinus was obliterated with fat and fibrin glue TISSEEL®. Histopathologic analysis of the biopsied tissue showed dark giant oval nuclei, with vacuolated or granular eosinophilic cytoplasm. The cells were arranged in epithelial cords, and on immunohistochemistry were strongly positive for S-100 protein and epithelial membrane antigen, thereby confirming diagnosis of a chordoma in the clival region.

**Figure 4:**
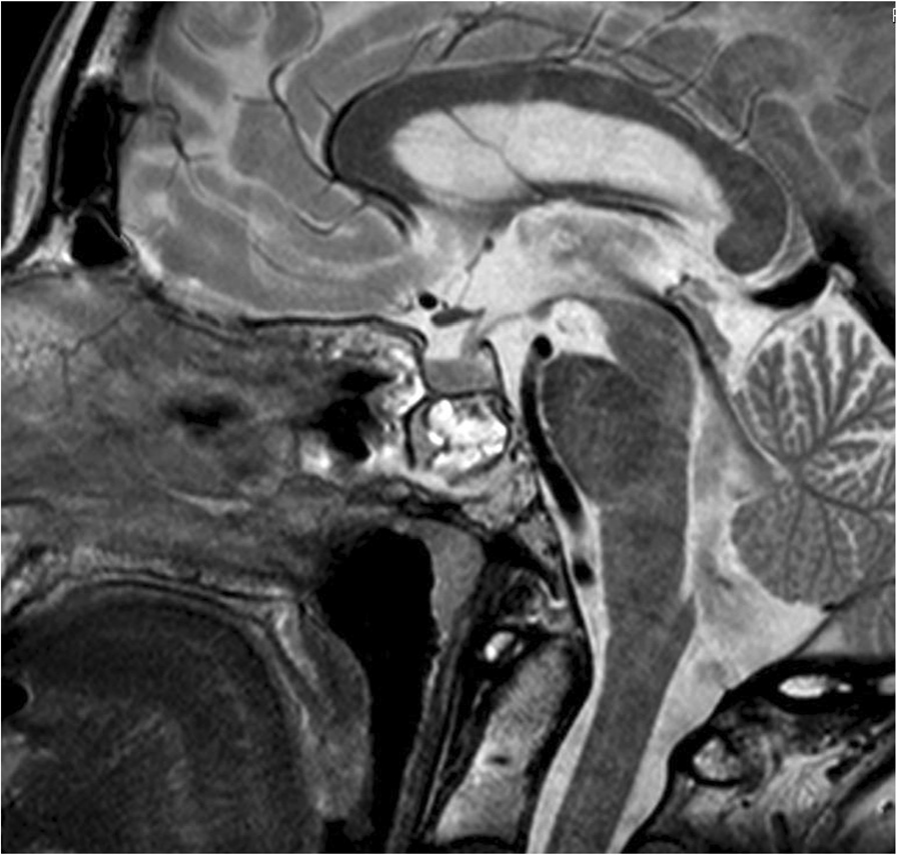
**T2-weighted sagittal images showing a predominantly hyperintense lesion involving the posterior wall of the clivus just anterior to the basilar artery.** The sphenoid sinus shows evidence of packing done during the first surgery with inflammatory changes.

**Figure 5:**
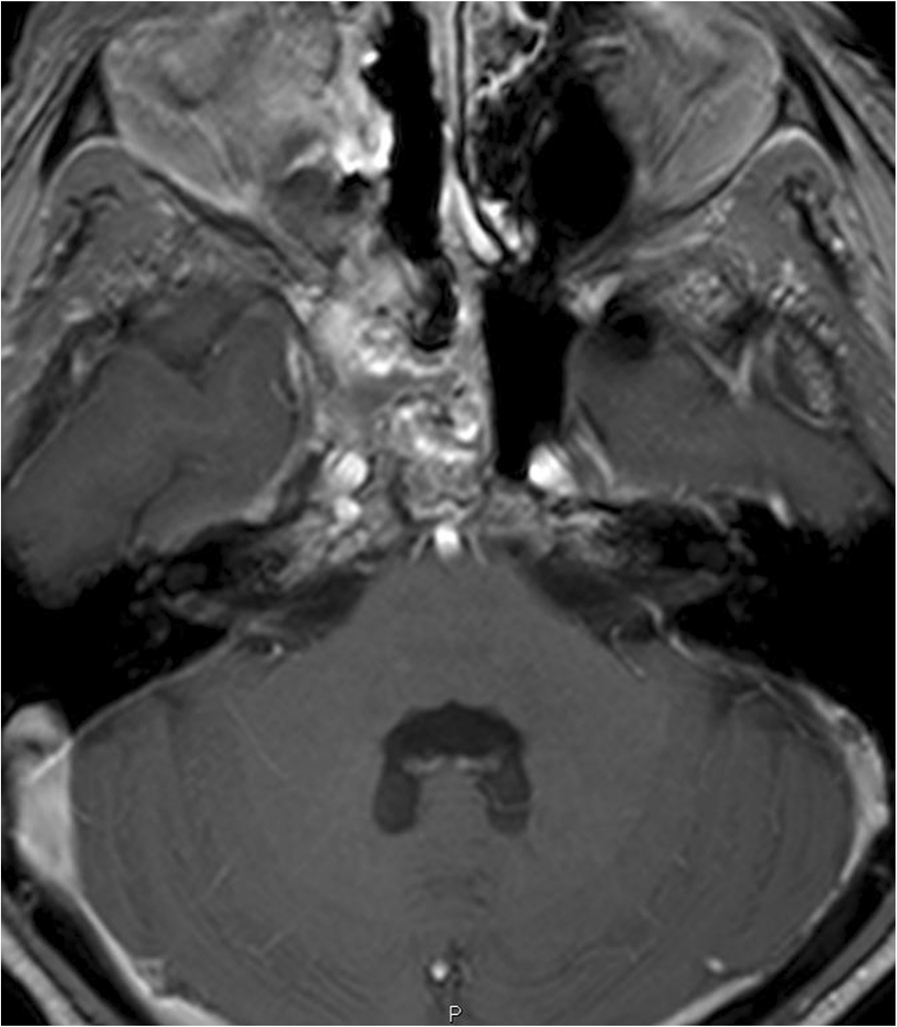
**T1-weighted gadolinium enhanced images show a hypointense lesion of the clivus more towards the right side with destruction of the bony margins of the clivus.** The lesion is found just anterior to the basilar artery. There was no contrast enhancement of the lesion.

**Figure 6:**
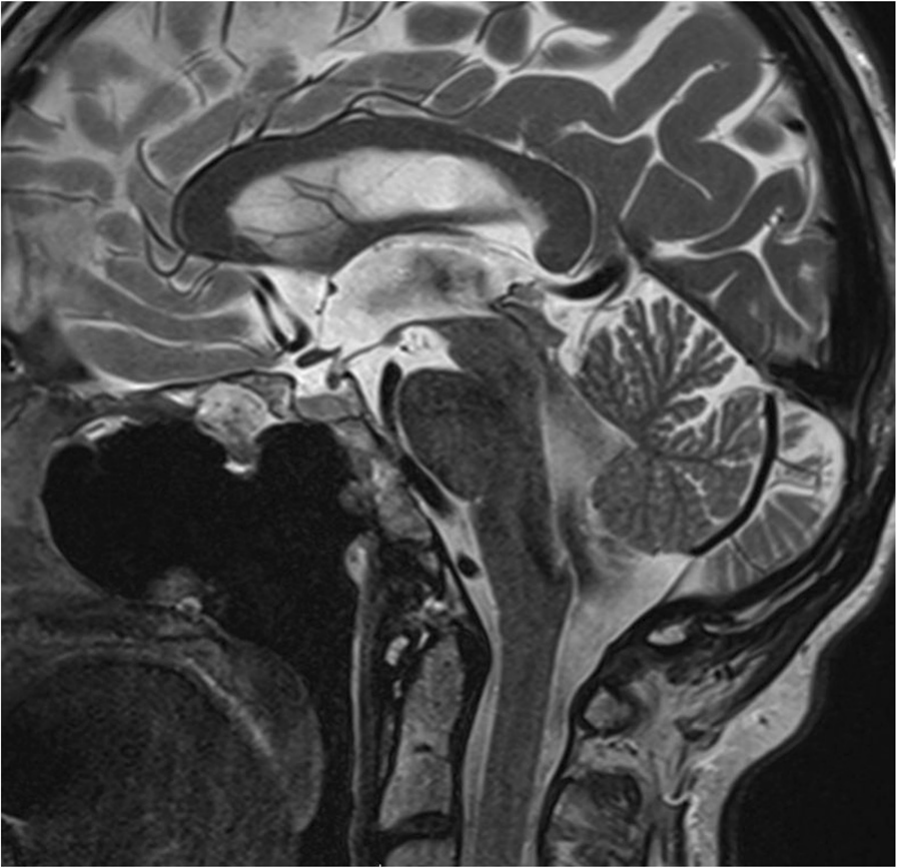
T2-weighted sagittal magnetic resonance scan shows total excision of the lesion with the packing material seen in the posterior part of the clivus in the extradural plane.

**Figure 7:**
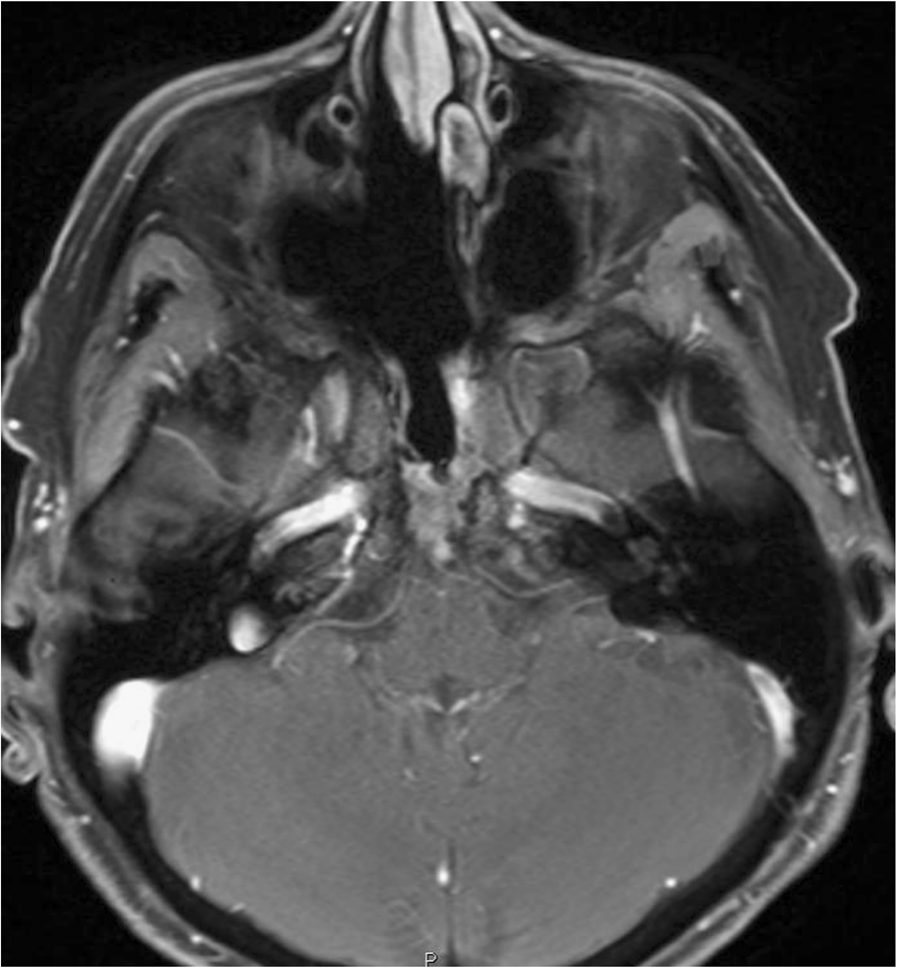
T1-weighted fat suppressed images show no evidence of residual or recurrent tumor.

Three months later, our patient underwent an MRI (Figures 4 and 5) to re-define the presence and extent of the clival chordoma. In the intervening period she was asymptomatic and did not have any CSF leak. A transnasal endoscopic resection of the chordoma aided by neuronavigation was planned. Surgery began with a complete right-side ethmoidectomy. Posterior septectomy allowed a 'two nostrils - four hands’ technique for tumor removal. A radical vomerectomy was performed. The entire anterior wall of her sphenoid sinus and her sphenoidal septum were excised. During a revision endoscopic exploration it is extremely important to identify the bony edges circumferentially around the earlier defect and gradually define the bone and soft tissue junction, which necessitated removal of the upper two-thirds of her clivus. The next step was to identify the sella, optic nerve and the carotid impressions bilaterally within her sphenoid sinus. Subsequently, the normal dura and fibrocicatricial tissue surrounding the chordoma were identified. An *en bloc* excision of the chordoma was done, anticipating her basilar artery to be just posterior to the tumor. Reconstruction of the defect was performed using fascia lata, fat and fibrin glue. A lumbar drain was inserted preoperatively and used to drain CSF in the postoperative period. It was removed on the fifth postoperative day. Broad spectrum antibiotics (ceftazidime 2g three times a day) were given for 10 days. Our patient had an excellent recovery with no complications. The follow-up scans showed complete excision of the tumor and no other adjuvant therapy was instituted. Our patient was discharged on the 12^th^ postoperative day and a systematic clinico-radiological follow-up is planned. She was totally asymptomatic at the latest follow-up (three months after surgery) with an MRI (Figures 6 and 7) showing complete excision of the tumor.

## Discussion

Chordomas were first described by Virchow in 1857 as tumors made up of vacuolated or ‘physaliferous’ cells derived from rests of embryonic notocord along the midline central nervous system axis [11]. Chordomas can invade and metastasize to bony structures like the sacrococcyx, skull base and vertebrae [11]. The histological appearance of a chordoma includes pleomorphic cells with dark nuclei and vacuolated or granular cytoplasm set within an abundant myxoid matrix. Neoplastic cells are arranged in epithelial cords separated by mucinous material, which is a classic feature of chordomas. On immunohistochemistry, the cells are positive for S-100 protein and epithelial membrane antigen [8]. The symptoms of a clival chordoma depend mainly on the site of the tumor and the adjacent structures. Headache, visual changes and cranial nerve palsies are the most frequent symptoms [4], though rare presentations like CSF rhinorrhea and epistaxis have also been reported [8,10].

For diagnosis, it is mandatory to do a CT scan and MRI for all skull base tumors, although there are no reliable diagnostic features that allow differentiation between these tumors [11]. Generally, MRI is better for defining the exact position of the brainstem and the optic chiasma relative to the tumor with added information about tumor extension into the nasopharynx and cavernous sinus. It also demonstrates the position of the cavernous internal carotid, vertebral and basilar arteries in relation to the tumor [12]. CT is better than MRI in demonstrating tumoral calcification and associated bone destruction.

Clival chordomas can be managed by a variety of conventional surgical approaches: transcranial, transsphenoidal, transoropharyngeal and maxillary osteotomy approaches [13]. Transcranial approaches involve brain retraction and have increased risks of cerebral edema and hematoma, apart from carotid, basilar artery and optic nerve trauma. These complications can be greatly reduced with anterior (transnasal, transoral and transfacial) approaches [13]. Currently, endoscopic surgery has opened a new avenue in the management of clival chordomas, not only as a direct surgical access but also by providing an excellent visualization of the clivus and surrounding structures, especially the anterior dura and the basilar artery. The surgical procedures, techniques and selection of the endoscopic surgical strategies for preservation of the vital anatomic structures are described in detail in the literature [13].

This case report is interesting on several counts. In general, patients with clival chordomas present with headache or cranial nerve palsies. Our patient was hospitalized as an emergency case with meningitis, which in turn was secondary to a CSF leak caused by skull base erosion. MRI showed an osteolytic, irregular lesion occupying her upper clivus that was hyperintense on T2-weighted images and hypointense on T1-weighted images, showing no enhancement with administration of gadolinium. Neither CT nor MRI images revealed any obvious tumor mass. Transnasal endoscopic exploration allowed us to close the skull base defect and collect local soft tissue for histological analysis, which showed evidence of a chordoma. A second surgery for an *en bloc* resection of the chordoma was performed three months after the initial intervention. It is quite possible that, right at the first presentation, our patient had a clival chordoma with skull base erosion, but it did not enhance on contrast administration and hence could not be diagnosed in the earlier CT and MRI scans. An intradural extension of the tumor led to a CSF leak and subsequent meningitis. A systematic exploration of her skull base allowed us to send local soft tissue for histological analysis, which confirmed the diagnosis of a chordoma and allowed us to plan the subsequent radical endoscopic excision.

A revision endoscopic skull base surgery can be difficult due to severe postoperative fibrocicatricial tissue as a result of the packing material (fat, fascia lata) used for the CSF leak closure and skull base reconstruction during the prior surgery. During such revision explorations, it is of paramount importance to define the tumoral soft tissue and bone erosion limits, followed by identification of the important landmarks within the sphenoid sinus. Neuronavigation helps in identifying vital anatomical structures at the anterior skull base and avoids intraoperative complications, thereby facilitating a complete tumor removal. The two nostrils - four hands technique facilitates better transnasal instrumentation allowing a complete tumor excision and efficient reconstruction of the skull base defect.

The role of radiation therapy has been extremely controversial in the treatment of clival chordomas. Conventional radiation does not appear to have an effect on survival in the study published by Colli and Al-Mefty [14]. Results of this study indicated that the patient overall survival significantly improved with a radical resection of the tumor combined with postoperative proton beam radiotherapy. There is no study in the literature comparing the results of surgery alone and surgery with postoperative radiotherapy. Finally, with a limited number of cases in the literature, we cannot conclude on the most effective and ideal management of these cases, though most authors agree that complete surgical excision of the tumor mass with an adequate margin provides the best chance for a recurrence-free survival [14,15].

## Conclusion

Patients with a clival chordoma commonly present with headaches, visual changes, cranial nerve palsies and, rarely, with CSF rhinorrhea and epistaxis. Patients who present with meningitis secondary to a CSF leak may have an underlying erosive lesion that can be missed on initial radiological examination. A revision transnasal endoscopic excision of a clival chordoma is challenging and warrants collaborative efforts between the ear, nose and throat surgeons and neurosurgeons.

## Consent

Written informed consent was obtained from the patient for publication of this manuscript and accompanying images. A copy of the written consent is available for review by the Editor-in-Chief of this journal.

## Competing interests

The authors declare that they have no competing interests.

## Authors’ contributions

JA - collection of data, manuscript writing. PM - analysis and editing. RTD - analysis and editing the manuscript. KS - collection of data, manuscript writing, analysis and editing the manuscript. KS was the major contributor in writing the manuscript. All authors read and approved the final manuscript.
